# Pre-stroke weight loss by glucagon-like peptide 1 receptor and neuropeptide Y receptor Y2 activation improves post-stroke functional recovery in male diabetic mouse models

**DOI:** 10.1007/s00125-025-06567-4

**Published:** 2025-10-15

**Authors:** Ellen Vercalsteren, Dimitra Karampatsi, Maria Neicu, Mihaela Oana Romanitan, Peter Haebel, Katherin Bleymehl, Thomas Nyström, Thomas Klein, Vladimer Darsalia, Cesare Patrone

**Affiliations:** 1 Department of Clinical Science and Education, Södersjukhuset, Internal Medicine, Karolinska Institutet, Stockholm, Sweden; 2 Boehringer Ingelheim Pharma GmbH & Co. KG, Biberach, Germany

**Keywords:** Glucagon-like peptide 1 receptor, Neuropeptide Y receptor Y2, Stroke, Type 2 diabetes, Weight loss

## Abstract

**Aims/hypothesis:**

Type 2 diabetes is associated with worsened stroke outcome and lasting disability. The underlying mechanisms are undetermined, and no therapy is available. We experimentally investigated whether pharmacologically targeting obesity, which is highly prevalent in type 2 diabetes, before stroke enhances neurological recovery in type 2 diabetes. To induce weight loss, we employed the glucagon-like peptide 1 receptor (GLP-1R) agonist semaglutide and the neuropeptide Y receptor Y2 (NPY2R) agonist BI8271, which potentiates GLP-1R-mediated weight loss. We also investigated potential acute neuroprotective effects induced by these treatments, independently of metabolic regulation.

**Methods:**

C57BL/6J mice were fed with a high-fat diet for 5 months to induce obesity and features of type 2 diabetes (i.e. hyperglycaemia and insulin resistance). Weight loss was induced by 4-week administration of semaglutide and/or BI8271. As a control for the effect of weight loss, a vehicle (PBS)-treated group was switched to standard diet to achieve the same weight range and the same percentage weight loss within the same time frame as those receiving semaglutide/BI8271. Thereafter, mice were subjected to stroke by transient middle cerebral artery occlusion (tMCAO). Stroke recovery (the primary outcome) was assessed by measuring the recovery of grip strength and the lateralised sensorimotor integration. Brains and serum were collected, and stroke volume and serum IGF-1 levels were quantified (secondary outcomes). In additional studies, type 2 diabetic mice were subjected to tMCAO and injected with semaglutide and/or BI8271 1 and 24 h after reperfusion. Acute neuroprotection (the primary outcome) was assessed by a grip strength test and by quantifying stroke volume and the number of surviving neuronal nuclear marker (NeuN)-positive neurons.

**Results:**

We report that pre-stroke weight loss by GLP-1R activation, and more potently by dual co-activation of GLP-1 and NPY2 receptors, is a pharmacologically targetable mechanism, upstream of glycaemic regulation, through which post-stroke recovery is achieved. Moreover, we show that post-stroke recovery in type 2 diabetes is inversely associated with peripheral IGF-1 levels. Finally, GLP-1R and NPY2R activation can also improve stroke recovery through acute neuroprotection if they are given acutely after stroke, independently of their metabolic effects.

**Conclusions/interpretation:**

The diabetes and obesity epidemics are increasing the incidence of stroke, and consequently the need for treatments to improve stroke outcome. Our results indicate that clinically used type 2 diabetes treatments could be employed in a preventive role to improve stroke outcome by exerting dual pharmacological action: weight loss and acute neuroprotection. These findings could have novel therapeutic implications for many people.

**Graphical Abstract:**

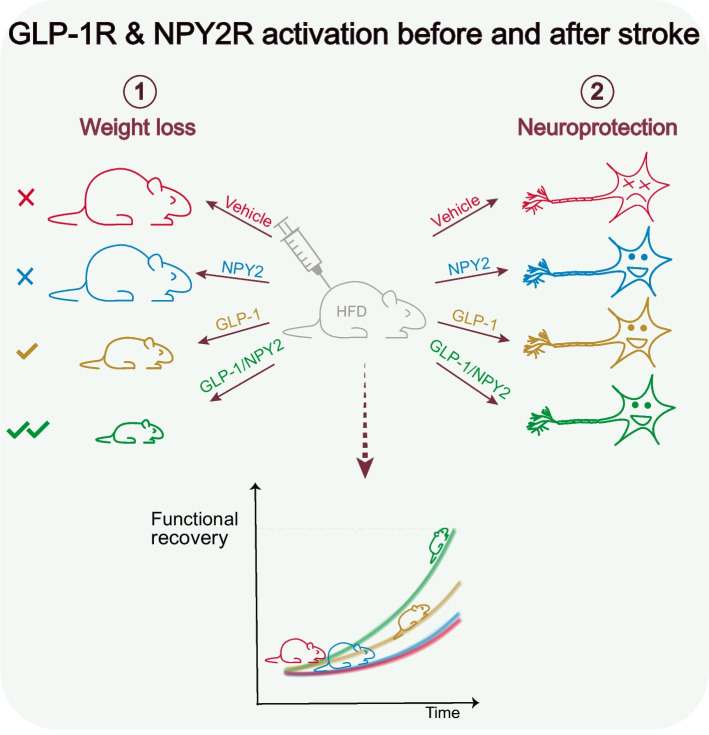

**Supplementary Information:**

The online version contains peer-reviewed but unedited supplementary material available at 10.1007/s00125-025-06567-4.


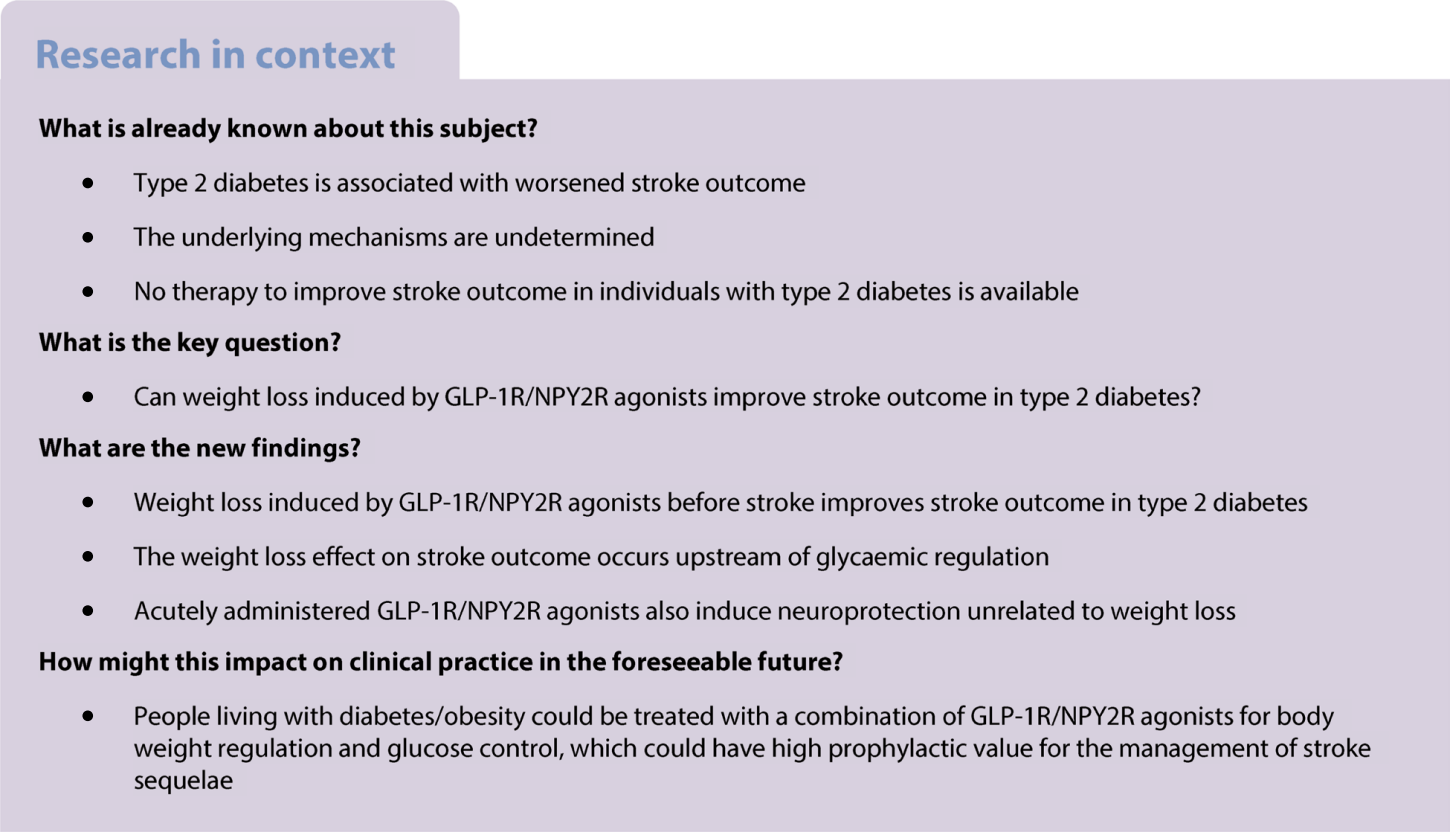


## Introduction

Type 2 diabetes [1] is one of the major risk factors for stroke [2, 3] and a strong predictor of lasting post-stroke disability [4–6]. In addition, overweight and obesity are highly prevalent in people living with type 2 diabetes [7], with more than 90% of them having a BMI ≥25.0 kg/m^2^ [8]. Obesity contributes to the pathology of many nervous system disorders, including stroke [9], and importantly increases the disability burden among stroke survivors with type 2 diabetes [10]. Weight loss in obesity/type 2 diabetes improves glucose metabolism, even leading to diabetes remission [11]. Furthermore, it reduces the incidence of cardiovascular disease, including stroke [12]. Therefore, targeting obesity in type 2 diabetes could also prove valuable to improve post-stroke neurological outcomes. However, this question has never been clinically addressed*.*

We previously showed in a mouse model of obesity/type 2 diabetes that diabetes remission induced by pre-stroke weight loss via a dietary intervention enhanced stroke recovery [13]. However, achieving weight loss solely by dietary and/or exercise interventions is very challenging for most individuals living with obesity [14]. Therefore, pharmacological targeting of obesity in type 2 diabetes could prove more effective, essentially by improving patient adherence and long-term weight management.

Glucagon-like peptide 1 receptor (GLP-1R) agonists such as semaglutide are widely used glucose-lowering drugs [15], which mainly decrease blood glucose levels by stimulating insulin release while suppressing glucagon secretion [16]. Interestingly, these drugs are also effective at reducing body weight [17, 18]. In addition, semaglutide reduces the risk of major adverse cardiovascular events in individuals with type 2 diabetes [19]. However, it remains unknown whether semaglutide-induced weight loss before stroke in type 2 diabetes can also improve post-stroke outcome.

We aimed to experimentally address this question by taking advantage of the effect of neuropeptide Y receptor Y2 (NPY2R) activation on food intake [20]. When co-activated with GLP-1R, NPY2R induces greater weight loss, improves insulin sensitivity and restores beta cell function in mouse models of obesity/type 2 diabetes [21–23]. We hypothesised that greater weight loss by co-activation of GLP-1R/NPY2R would have a greater effect on post-stroke recovery than semaglutide monotherapy. Furthermore, we investigated potential associations between improved post-stroke recovery and serum IGF-1 levels, as IGF-1 has been shown to be associated with post-stroke functional outcome [24, 25]. Finally, to investigate whether semaglutide monotherapy or semaglutide in combination with BI8271, an NPY2R agonist (see electronic supplementary material [ESM] Methods: Diets and compounds), could also increase post-stroke neuronal survival, independently of metabolic effects, we determined their acute neuroprotective potential.

## Methods

For detailed methods, please refer to ESM Methods.

### Effect of pre-stroke weight loss on stroke recovery (studies 1 and 2)

**Fig. 1:**
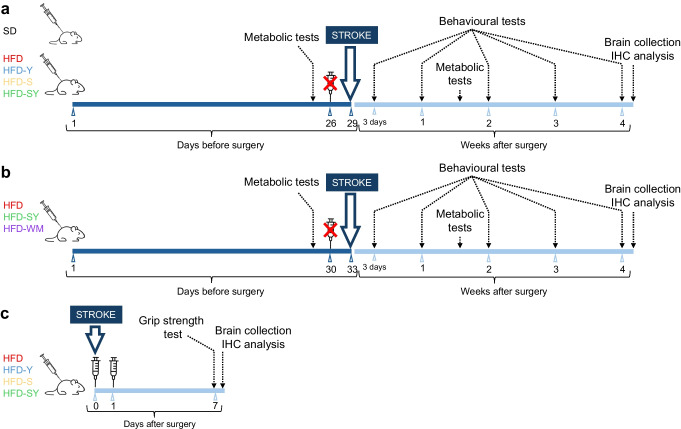
Experimental design. Male C57BL/6J mice were fed with HFD for 5 months to induce obesity and diabetic features. (**a**) Study 1. Effect on stroke recovery of weight loss that was pharmacologically induced by semaglutide monotherapy (HFD-S), BI8271 monotherapy (HFD-Y) or a combination of semaglutide and BI8271 (HFD-SY). Briefly, mice were treated daily for 26 days. Treatments were withdrawn 2 days prior to stroke surgery to avoid potential acute neuroprotective effects. At day 29, experimental stroke was induced by tMCAO. After tMCAO, all mice were switched to a standard diet, and metabolic and behavioural tests were performed before collecting their brains for immunohistochemical (IHC) analysis. (**b**) Study 2. Effect on stroke recovery of diet-based weight loss vs weight loss that was pharmacologically induced by a combination of semaglutide and BI8271. Diabetic mice were randomised into one of three groups: (1) a group that was maintained on HFD without intervention (HFD); (2) a group that was switched to standard diet (HFD-WM); and (3) a group that continued on HFD but was injected with semaglutide and BI8271 daily (HFD-SY). Animals were treated for 30 days. Treatments were withdrawn 2 days prior to stroke to avoid potential acute neuroprotective effects. At day 33, mice were subjected to tMCAO. After tMCAO, all mice were switched to a standard diet, and metabolic and behavioural tests were performed weekly prior to brain collection for IHC analysis. (**c**) Study 3. Neuroprotective effects of semaglutide monotherapy, BI8271 monotherapy and the combination of semaglutide and BI8271 on stroke-induced brain damage. Stroke was induced by tMCAO and thereafter all mice were switched to a standard diet. Mice were then injected s.c. at 1 and 24 h after stroke. Behavioural tests were performed 1 week after stroke, prior to brain collection for IHC analysis. SD, Standard diet

In study 1, 100 male mice (4 weeks old) were kept for 5 months on either a high-fat diet (HFD) (*n*=80) or standard diet (SD; *n*=20) (see ESM Methods: Diets and compounds). When the mice on the HFD became obese (≥50% weight gain compared with lean control mice) with diabetic features (fasting glucose >7 mmol/l and insulin resistance), they were randomly allocated to one of four groups and subjected to 26 days of daily s.c. injections (in the evening) with (1) semaglutide (3 nmol/kg) (HFD-S, *n*=20); (2) BI8271 (3 nmol/kg) (HFD-Y, *n*=20); (3) semaglutide (3 nmol/kg) in combination with BI8271 (3 nmol/kg) (HFD-SY, *n*=19); or (4) vehicle (PBS) only (HFD, *n*=19) (see ESM Methods: Diets and compounds, for details of the pharmacological treatments). Body weight was measured daily to monitor weight loss, and the glucose-lowering efficacy of the treatments was determined after 26 days by measuring hyperglycaemia, hyperinsulinaemia and insulin resistance (see ESM Methods: Metabolic tests). After a 2 day washout period, mice were subjected to either 35 min transient middle cerebral artery occlusion (tMCAO; see ESM Methods: Transient middle cerebral artery occlusion) (*n*=14 or 15 per group) or sham surgery (*n*=5 per group). Stroke recovery (the primary outcome) was monitored by behavioural tests (see ESM Methods: Assessment of stroke recovery): (1) a grip strength test, starting at 3 days after tMCAO, and weekly for 4 weeks, and (2) a corridor test, performed at weeks 1 and 4 after tMCAO (Fig. 1a and ESM Fig. 1a). Then the brains and serum were collected (see ESM Methods: Immunohistochemistry) for analysis of secondary outcomes (stroke volume, number of neurons positive for the neuronal nuclear marker [NeuN] and serum IGF-1 levels, see ESM Methods: Ischaemic stroke volume assessment and ESM Methods: ELISA immunoassays).

In study 2, we assessed whether improved stroke recovery by semaglutide and BI8271 occurred exclusively via weight loss by comparing stroke recovery between two groups of mice in which the same weight loss was achieved by either changing the HFD to standard diet or by a pharmacological intervention with semaglutide plus BI8271. To this end, diabetic features were induced in 35 male mice at 4 weeks old, as described above. Then mice were randomised into three groups: (1) a group kept on HFD and treated with semaglutide plus BI8271 (HFD-SY, *n*=12) as described above; (2) a vehicle (PBS)-treated group that was switched to standard diet to achieve the same weight range and the same percentage weight loss within the same time frame as in the semaglutide/BI8271 group (HFD-WM, *n*=12); and (3) a vehicle (PBS)-treated group that continued on HFD (HFD, *n*=11). After 30 days, when mice in both the HFD-SY and HFD-WM groups reached the weight range of lean age-matched control mice, treatment was withdrawn and the glucose-lowering efficacy of the interventions was determined by measuring hyperglycaemia, hyperinsulinaemia and insulin resistance. After a 2 day washout period, mice were subjected to tMCAO as described above. Stroke recovery was monitored as described in study 1. At the end of the experiment, brains were collected for histological analysis of stroke volume (Fig. 1b and ESM Fig. 1b).

To assess the acute glycaemic effect of the treatments, 30 additional 4-week-old male mice were kept on HFD for 5 months to induce obesity/diabetic features, as described above. Mice were then fasted for 1 h and baseline blood glucose was measured. Then, mice were randomly allocated to one of four groups and injected s.c. with (1) semaglutide (3 nmol/kg) (*n*=7); (2) BI8271 (3 nmol/kg) (*n*=7); (3) semaglutide (3 nmol/kg) in combination with BI8271 (3 nmol/kg) (*n*=8); or (4) vehicle (PBS) (*n*=8), and glucose was measured at 1, 2, 3 and 4 h thereafter.

### Acute neuroprotection post-stroke (study 3)

Starting at 4 weeks of age, 40 male mice were put on an HFD for 5 months to induce obesity and diabetic features, as described above. Then mice were subjected to tMCAO. The animals were randomly allocated to one of four groups 1 h after reperfusion and given a 3 nmol/kg bolus s.c. injection of (1) semaglutide (*n*=10); (2) BI8271 (*n*=10); (3) semaglutide in combination with BI8271 (*n*=10); or (4) vehicle (PBS) (*n*=10). An additional bolus treatment was given the day after stroke. Neuroprotection (the primary outcome) was assessed 1 week after tMCAO by a grip strength test, and, after the brains were collected, by measuring stroke volume and by stereological quantification of surviving NeuN-positive neurons throughout striatum/cortex (Fig. 1c and ESM Fig. 1c).

### Statistical analysis

Details of the statistical analysis methods are given in ESM Methods: Statistical analysis. Calculation of the necessary sample sizes is described in ESM Methods: Sample size calculation. Some animals were removed in each study (see ESM Methods: Animals before and after exclusion, and ESM Fig. 1a–c).

## Results

### Semaglutide induces weight loss and improves glucose metabolism in a mouse model of diabetes; effects potentiated by BI8271

**Fig. 2:**
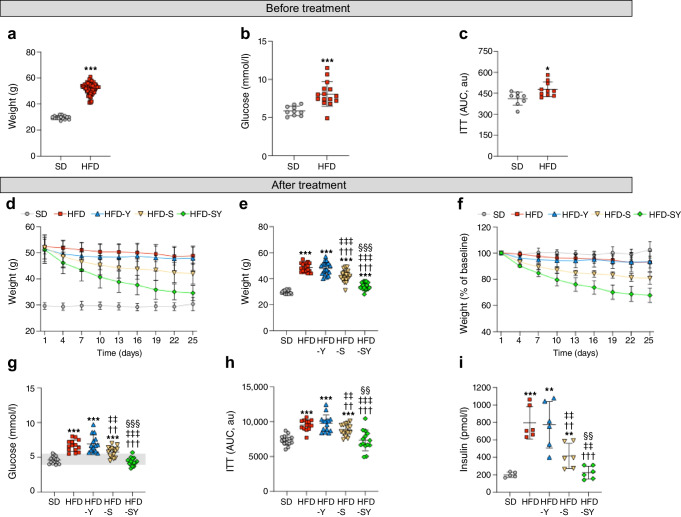
Effects of semaglutide monotherapy, BI8271 monotherapy and the combination of semaglutide and BI8271 on weight and glucose metabolism before stroke (study 1). (**a**–**c**) Pre-treatment effect of HFD feeding on body weight (**a**), fasting glucose (**b**) and insulin sensitivity (**c**). (**d**–**i**) Effect of semaglutide and BI8271 treatment on body weight (**d**–**f**), fasting glucose (**g**), insulin sensitivity (**h**) and fasting insulin (**i**). Data are presented as mean ± SD. The grey shaded area in (**g**) indicates the normoglycaemic range. Statistical analyses were performed using Welch’s test (**a**–**c**) or the Brown–Forsythe test and Welch ANOVA followed by the two-stage linear step-up procedure of Benjamini, Krieger and Yekutieli (**e**–**i**): *p<0.05 vs SD; **^/††/‡‡/§§^*p*<0.01 vs SD, HFD, HFD-Y and HFD-S, respectively; ***^/†††/‡‡‡/§§§^*p*<0.001 vs SD, HFD, HFD-Y and HFD-S, respectively. Sample sizes: (**a**) SD, *n*=20; HFD, *n*=78; (**b**) SD, *n*=10; HFD, *n*=15 (representative sample of mice, randomly selected); (**c**) SD, *n*=8; HFD, *n*=10 (representative sample of mice, randomly selected); (**d**–**f**) SD, *n*=20; HFD, *n*=19; HFD-Y, *n*=20; HFD-S, *n*=20; HFD-SY, *n*=19; (**g**, **h**) *n*=14–15 per group (representative sample of mice, randomly selected); (**i**) *n*=5–6 per group (each datapoint represents results from pooled serum samples from 2 or 3 mice). au, arbitrary units. SD, Standard diet

In study 1, prolonged exposure to HFD (5 months) induced obesity, fasting hyperglycaemia and insulin resistance (Fig. 2a–c). To induce weight loss, the mice were then treated for 4 weeks with semaglutide monotherapy or in combination with BI8271. The rest of the mice received BI8271 or vehicle (PBS) treatment (Fig. 1a).

Semaglutide gradually reduced body weight, and this effect was potentiated by BI8271 (Fig. 2d). Four weeks after the start of treatment, semaglutide monotherapy reduced body weight by approximately 20% (Fig. 2e, f) and semaglutide in combination with BI8271 reduced body weight by approximately 33% (Fig. 2e, f), reaching weights similar to those of standard diet-fed mice (Fig. 2e). BI8271 monotherapy was completely ineffective in reducing body weight (Fig. 2d–f).

Semaglutide monotherapy also significantly improved fasting glucose (Fig. 2g), insulin sensitivity (Fig. 2h) and fasting insulin levels (Fig. 2i) compared to HFD mice treated with BI8271 and HFD mice treated with vehicle (PBS), although the levels of these metabolic parameters remained significantly higher in comparison with mice fed a standard diet (Fig. 2g–i). In contrast, treatment of HFD mice with semaglutide in combination with BI8271 completely normalised fasting glucose, insulin resistance and fasting insulin to the same levels as in mice fed a standard diet (Fig. 2g–i).

### Semaglutide improves neurological recovery after stroke in a mouse model of diabetes; effect synergised by BI8271

**Fig. 3:**
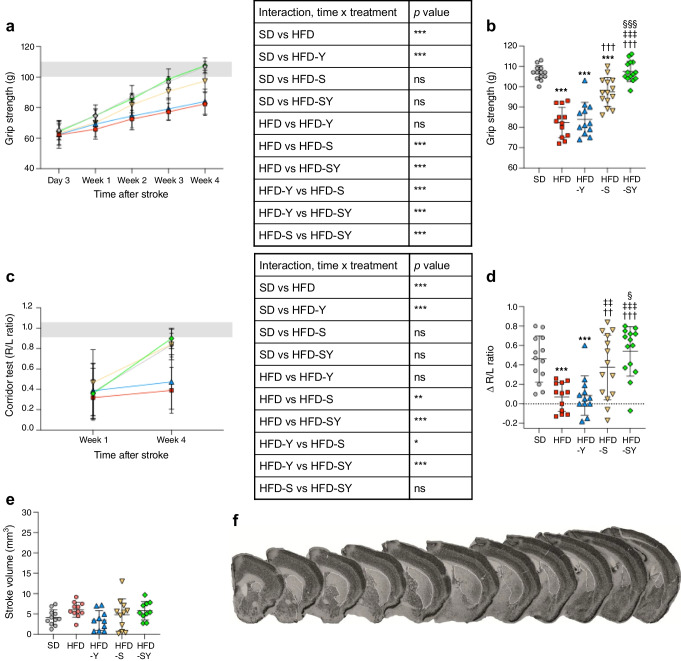
Effects of treatment before stroke with semaglutide monotherapy, BI8271 monotherapy or the combination of semaglutide and BI8271 on functional recovery after stroke (study 1). (**a**, **b**) Forepaw grip strength during stroke recovery shown as a plotted curve with the corresponding ANOVA table (**a**), and the grip strength at 4 weeks after stroke (**b**). (**c**, **d**) Results of the corridor test at 1 and 4 weeks post-stroke shown as a plotted curve with the corresponding ANOVA table (**c**), and the increase in R/L ratio between week 4 and week 1 (**d**). The grey shaded areas in (**a**) and (**c**) indicate the range of pre-stroke grip strength (**a**) and R/L ratio (**c**). (**e**, **f**) Ischaemic stroke volume (**e**) and representative images for NeuN staining (**f**). The white dotted lines indicate the stroke area. Data are presented as mean ± SD. Statistical analyses were performed using two-way repeated measures ANOVA (**a**, **c**), the Brown–Forsythe test and Welch ANOVA followed by the two-stage linear step-up procedure of Benjamini, Krieger and Yekutieli (**b**, **d**) or ordinary one-way ANOVA followed by the two-stage linear step-up procedure of Benjamini, Krieger and Yekutieli (**e**): ^§^*p*<0.05 vs HFD-S; ^††/‡‡^*p*<0.01 vs HFD and HFD-Y, respectively; ***^/†††/‡‡‡/§§§^*p*<0.001 vs SD, HFD, HFD-Y and HFD-S, respectively. Sample sizes: (**a**–**d**) SD, *n*=13; HFD, *n*=12; HFD-Y, *n*=12; HFD-S, *n*=14; HFD-SY, *n*=14; (**e**) SD, *n*=11; HFD, *n*=11; HFD-Y, *n*=10; HFD-S, *n*=11; HFD-SY, *n*=10. ns, not significant. SD, Standard diet

Three days after the last drug treatment, mice were tested for baseline grip strength and subjected to tMCAO or sham surgery. From day 3 after tMCAO, neurological recovery was tracked by weekly grip strength tests for 4 weeks (study 1, Fig. 1a). Baseline grip strength was similar in all groups (ESM Fig. 2). At 3 days after tMCAO, all mice showed approximately 45% reduction in their right forepaw grip strength (Fig. 3a). During the 4-week recovery period, HFD-induced type 2 diabetes significantly impaired the recovery of forepaw grip strength compared with mice fed a standard diet, leading to significantly lower grip strength at 4 weeks post-stroke (Fig. 3b). Treatment with the NPY2R agonist BI8271 showed no improvement of either grip strength recovery (Fig. 3a) or endpoint grip strength compared with HFD mice (Fig. 3b). Semaglutide treatment resulted in significantly improved grip strength recovery compared with HFD mice, and significantly higher grip strength at endpoint (Fig. 3b). However, the endpoint grip strength remained significantly lower compared with mice fed a standard diet (Fig. 3b). In contrast, the combination of semaglutide and BI8271 not only significantly improved grip strength recovery and endpoint grip strength compared with semaglutide monotherapy (Fig. 3a, b), but also fully normalised both grip strength recovery and endpoint grip strength to values similar to those in mice fed a standard diet (Fig. 3a, b).

The recovery of the lateralised sensorimotor integration after tMCAO was evaluated by performing a corridor test. At 1 week after tMCAO, all mice showed strong left-side bias (right/left [R/L] ratio of approximately 0.4; Fig. 3c). At 4 weeks, HFD mice showed a stronger left-side bias compared with mice fed a standard diet (Fig. 3c, d), confirmed by a lack of increase in R/L ratio at week 4 compared with week 1 (difference of approximately 0.07; Fig. 3d). Similarly to HFD mice, mice treated with the NPY2R agonist BI8271 showed no significant improvement after 4 weeks compared with mice fed a standard diet (Fig. 3c, d). Both semaglutide alone or in combination with BI8271 significantly improved R/L lateralisation compared with HFD mice (differences of approximately 0.4 and approximately 0.5, respectively, Fig. 3c, d), with both groups reaching the levels of mice fed a standard diet. Interestingly, the increase in R/L ratio was significantly higher in the combination treatment group compared with semaglutide monotherapy, suggesting a stronger effect of the combination therapy in improving stroke recovery.

No differences in stroke-induced brain damage were observed between the groups (Fig. 3e and ESM Fig. 3).

To determine whether post-stroke weight loss influenced post-stroke recovery in type 2 diabetes, we also monitored body weight after stroke. Some initial weight loss was observed within the first week after stroke in the HFD, HFD-Y and HFD-S groups. However, from day 7 until they were killed, no differences between HFD groups were observed (ESM Fig. 4). Thus, we conclude that post-stroke weight loss has minimal/no effect on stroke recovery.

These results suggest that pre-stroke normalisation of body weight/glucose metabolism, either partially via semaglutide monotherapy or completely, via semaglutide and BI8271, improves post-stroke functional recovery without affecting stroke volume. This effect is stronger after the complete normalisation of body weight/glucose metabolism.

### Stroke recovery by semaglutide plus BI8271 is driven by weight loss

**Fig. 4:**
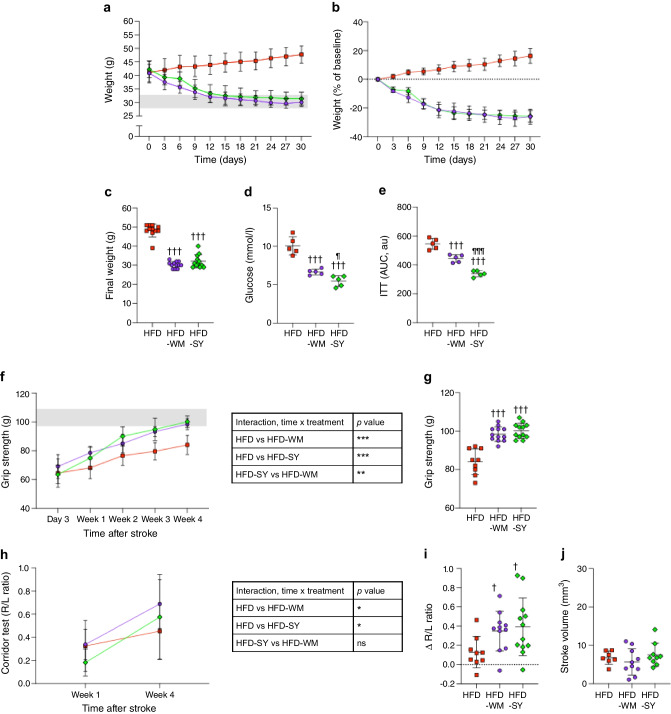
Effect of pre-stroke diet-based weight loss vs weight loss that was pharmacologically induced using semaglutide and BI8271 on functional recovery after stroke (study 2). (**a**–**c**) Effect of pre-stroke diet switch (from HFD to standard diet) or the combination of semaglutide and BI8271 on body weight, shown in terms of weight (**a**), percentage weight loss (**b**) and final body weight (**c**). (**d**, **e**) Effects on fasting glucose (**d**) and insulin sensitivity (**e**). (**f**, **g**) Forepaw grip strength during stroke recovery shown as a plotted curve with the corresponding ANOVA table (**f**), and the grip strength at 4 weeks after stroke (**g**). (**h**, **i**) Results of the corridor test at 1 and 4 weeks post-stroke shown as a plotted curve with the corresponding ANOVA table (**h**), and increase in R/L ratio between week 4 and week 1 (**i**). (**j**) Ischaemic stroke volume. Data are presented as mean ± SD. The grey shaded area in (**a**) indicates the weight range of lean, age-matched control mice, and that in (**f**) indicates the range of pre-stroke grip strength. Statistical analyses were performed using two-way repeated measures ANOVA (**a**, **c**, **f**, **h**) or the Brown–Forsythe test and Welch ANOVA followed by the two-stage linear step-up procedure of Benjamini, Krieger and Yekutieli (**b**, **d**, **e**, **g**, **i**, **j**): ^†/¶^*p*<0.05 vs HFD and HFD-WM, respectively; ^†††/¶¶¶^*p*<0.001 vs HFD and HFD-WM, respectively. Sample sizes: (**a**–**c**) HFD, *n*=11; HFD-WM, *n*=12; HFD-SY, *n*=12; (**d**, **e**) *n*=5 per group; (**f**) HFD, *n*=9; HFD-WM, *n*=12; HFD-SY. *n*=12; (**g**–**i**) HFD, *n*=9; HFD-WM, *n*=12; HFD-SY, *n*=12; (**j**) HFD, *n*=7; HFD-WM, *n*=10; HFD-SY, *n*=9. au, arbitrary units; ns, not significant

To determine whether improved stroke recovery by treatment with semaglutide/BI8271 was indeed due to weight loss, we compared stroke recovery between mice that were treated with semaglutide plus BI8271 and a group that was weight-matched to achieve the same weight loss by swapping HFD to standard diet for 30 days (study 2, Fig. 1b). Both interventions induced similar weight loss (Fig. 4a–c) and normalised diabetic features, with the combined semaglutide plus BI8271 treatment having a more pronounced effect (Fig. 4d, e). Importantly, both interventions improved functional recovery after stroke to a similar extent (Fig. 4f–i), without affecting infarct size (Fig. 4j), indicating that enhanced stroke recovery by GLP-1R and NPY2R activation occurs entirely via weight loss.

**Fig. 5:**
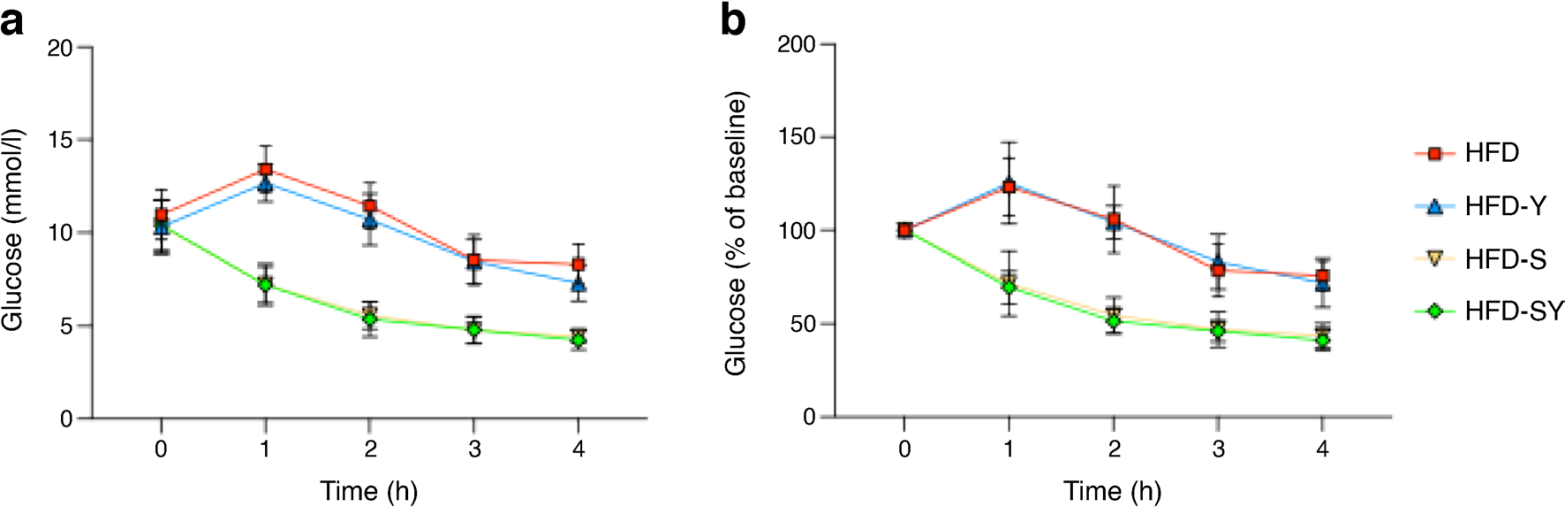
Effect of acute semaglutide monotherapy, BI8271 monotherapy and the combination of semaglutide and BI8271 on glucose in HFD-fed mice (study 3). Blood glucose levels after acute treatment shown as a plotted curve (**a**) and presented as percentage of glucose from baseline for each group (**b**). Sample sizes: HFD, *n*=8; HFD-Y, *n*=7; HFD-S, *n*=7; HFD-SY, *n*=8

To further investigate whether stronger stroke recovery after treatment with semaglutide in combination with BI8271 compared with semaglutide monotherapy was due to stronger effects on pre-stroke weight loss or glucose regulation per se (both of which were more potently regulated by the combination of semaglutide with BI8271), we assessed potential differences in acute glucose regulation in response to these two treatments, while weight remained unchanged. HFD mice were acutely given either semaglutide monotherapy or semaglutide in combination with BI8271, and glucose was measured hourly for a total of 4 h. The groups of mice receiving semaglutide monotherapy and those treated with semaglutide in combination with BI8271 both reached normoglycaemia (approximately 4 mmol/l) after 4 h, but mice treated with vehicle (PBS) or those receiving BI8271 monotherapy remained hyperglycaemic (>7 mmol/l) (Fig. 5a, b).

These results indicate that the superior effect of the combination of semaglutide and BI8271 compared with semaglutide monotherapy to regulate glucose after 4 weeks of treatment is secondary to the induction of weight loss. In turn, this indicates that a stronger pre-stroke weight loss (leading to stronger glycaemic regulation)—rather than glycaemic regulation per se—is a causal factor in improving post-stroke recovery in type 2 diabetes.

### Peripheral levels of IGF-1 both pre-stroke and in the sub-acute phases after stroke are inversely correlated with post-stroke recovery

**Fig. 6:**
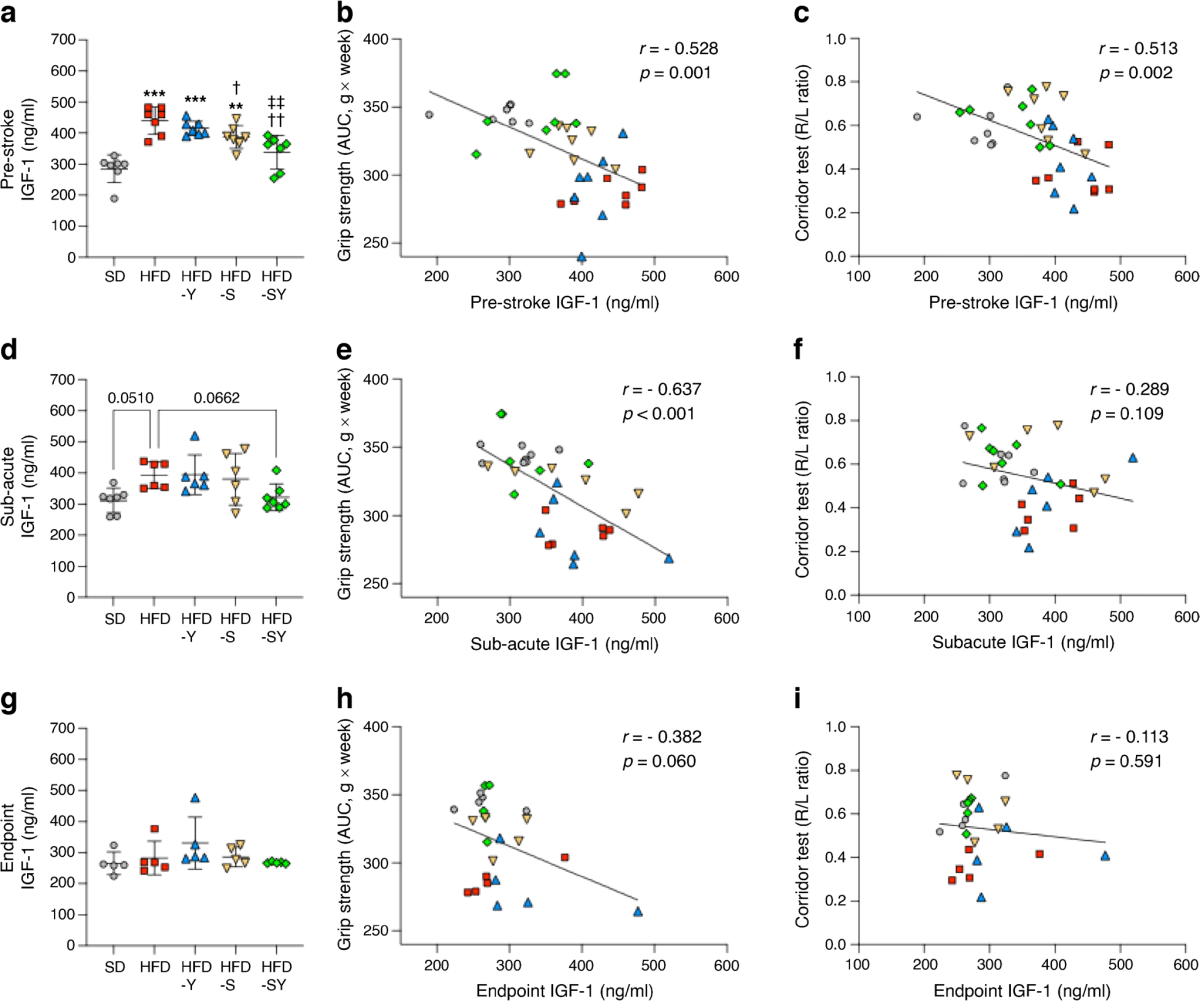
Effect of semaglutide monotherapy, BI8271 monotherapy and the combination of semaglutide and BI8271 on peripheral IGF-1 levels and their correlation with stroke recovery. (**a**–**c**) Pre-stroke IGF-1 levels between the treatment groups (**a**), and their correlation with grip strength (**b**) and R/L lateralisation (**c**). (**d**–**f**) IGF-1 levels in the sub-acute phase (at day 10 after tMCAO) (**d**), and their correlation with grip strength (**e**) and R/L lateralisation (**f**). (**g**–**i**) IGF-1 levels at endpoint (at 4 weeks after stroke) (**g**), and their correlation with the grip strength (**h**) and R/L lateralisation (**i**). Data are presented as mean ± SD. Statistical analyses were performed using the Brown–Forsythe test and Welch ANOVA followed by the two-stage linear step-up procedure of Benjamini, Krieger and Yekutieli (**a**, **d**, **g**) or Pearson correlation (**b**, **c**, **e**, **f**, **h**, **i**): ^†^*p*<0.05 vs HFD; **^/††/‡‡^*p*<0.01 vs SD, HFD and HFD-Y, respectively; ***p<0.001 vs HFD. Sample sizes: (**a**–**c**) SD, *n*=7; HFD, *n*=7; HFD-Y, *n*=7; HFD-S, *n*=7; HFD-SY, *n*=7; (**d**–**f**) SD, *n*=7; HFD, *n*=6; HFD-Y, *n*=6; HFD-S, *n*=6; HFD-SY, *n*=7; (**g**–**i**) SD, *n*=5; HFD, *n*=5; HFD-Y, *n*=5; HFD-S, *n*=5; HFD-SY, *n*=5. Each datapoint in this figure represents the results from pooled serum samples from 2 or 3 mice. SD, Standard diet

Decreased levels of IGF-1 have been shown to be associated with better functional outcome after stroke [24]. To determine the potential association between IGF-1 and stroke recovery in our study, we quantified serum IGF-1 levels in the pre-stroke, sub-acute (at 10 days post-stroke) and endpoint phases after stroke (Fig. 6). We show that HFD-fed mice treated with either vehicle (PBS) or BI8271 had significantly higher IGF-1 levels pre-stroke compared with mice fed a standard diet (Fig. 6a). Treatment with semaglutide reduced pre-stroke IGF-1 levels compared with HFD-fed vehicle (PBS)-treated mice, but levels remained higher than in mice fed a standard diet (Fig. 6a). However, treatment with semaglutide in combination with BI8271 resulted in similar IGF-1 levels to those in mice fed a standard diet (Fig. 6a). Interestingly, pre-stroke IGF-1 levels were significantly and inversely correlated with grip strength recovery and with R/L lateralisation, showing that lower pre-stroke IGF-1 levels were associated with enhanced post-stroke recovery (Fig. 6b, c). Similarly, in the sub-acute phase after stroke, IGF-1 levels in HFD mice remained higher compared with those in mice fed a standard diet (*p*=0.051), while treatment with semaglutide in combination with BI8271 resulted in levels that were similar to those in mice fed a standard diet (Fig. 6c). The sub-acute IGF-1 levels (Fig. 6d) were significantly and inversely correlated with the grip strength recovery (Fig. 6e), and a strong but not significant trend in the same direction was observed for R/L lateralisation (Fig. 6f). At 4 weeks post-stroke, significant differences in IGF-1 levels were not observed between groups (Fig. 6g) and there was no correlation with stroke recovery (Fig. 6h, i).

We conclude that increased peripheral IGF-1 levels during the sub-acute phase after stroke are directly associated with impaired stroke recovery in HFD-fed mice, and that this effect can be mitigated by GLP-1R activation and completely restored to the levels observed in mice fed a standard diet by co-activation of GLP-1R and NPY2R.

### Effect of semaglutide and BI8271 on acute neuroprotection

Next, we determined the potential acute neuroprotective efficacy of semaglutide, BI8271 and the combination of semaglutide and BI8271, independently of weight and glucose metabolism regulation (study 3). Specifically, we administered a 3 nmol/kg bolus of BI8271, semaglutide or a combination of semaglutide and BI8271 to HFD mice at 1 and 24 h after reperfusion, and the mice were killed 1 week thereafter (Fig. 1c).

**Fig. 7:**
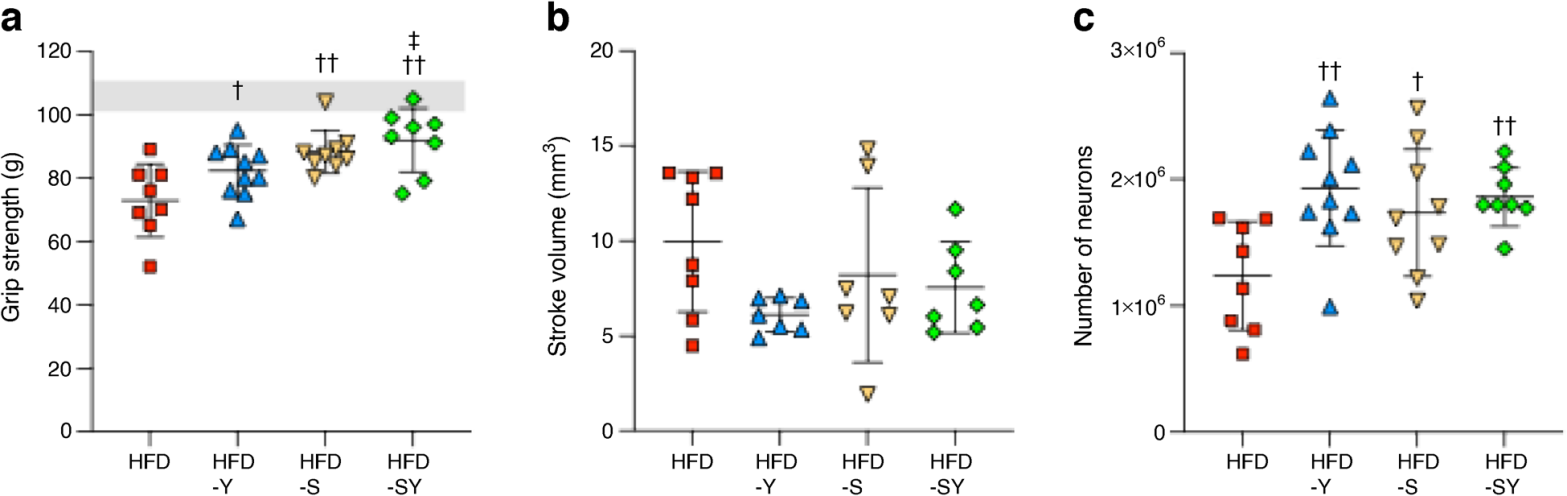
Effect of acute treatment with semaglutide, BI8271 and the combination of semaglutide and BI8271 on stroke-induced brain damage/neuroprotection (study 3). (**a**) Forepaw grip strength at 1 week after tMCAO. The grey shaded area indicates the range of pre-stroke grip strength. (**b**) Ischaemic stroke volume at 1 week after tMCAO. (**c**) Number of surviving NeuN-positive neurons at 1 week after tMCAO. Data are presented as mean ± SD. Statistical analyses were performed using one-way ANOVA followed by the two-stage linear step-up procedure of Benjamini, Krieger and Yekutieli. ^†/‡^*p*<0.05 vs HFD and HFD-Y, respectively; ^††^*p*<0.01 vs HFD. Sample sizes: HFD, *n*=8; HFD-Y, *n*=7–10; HFD-S, *n*=7–9; HFD-SY, *n*=7–8

Notably, all treatments significantly improved grip strength compared with HFD-fed control mice 7 days post-stroke (Fig. 7a). In addition, all treatments induced a non-statistically significant trend towards the reduction of stroke volume in comparison with HFD-fed control mice (Fig. 7b). These trends motivated us to quantify the number of surviving neurons (based on the number of NeuN-positive neurons) using a computerised stereology set-up and optical fractionator method [26]. The results show a significantly higher number of surviving neurons in all three treatment groups compared with HFD control mice (Fig. 7c).

We conclude that GLP-1R and NPY2R activation alone or in combination exhibit additional neuroprotective effects when administered acutely after stroke.

## Discussion

This study demonstrates experimentally that pre-stroke pharmacological targeting of obesity by GLP-1R and NPY2R activation in type 2 diabetes enhances stroke recovery, without effects additional to weight loss mediated by the treatments. Importantly, we show that weight loss is upstream of glucose regulation in terms of improvements in stroke recovery. Finally, we show that the same pharmacological strategies that are efficacious in improving stroke recovery through pre-stroke weight loss (via GLP-1R/NPY2R) can additionally provide acute neuroprotective effects post-stroke, independently of weight loss induction.

Type 2 diabetes doubles the risk of permanent disability after stroke, severely affecting patients’ quality of life [4–6]. However, despite the majority of individuals with type 2 diabetes being overweight or obese [7, 8], no clinical study has investigated the possibility of preventing disability after stroke in type 2 diabetes by normalising body weight before stroke.

We addressed this question by using the GLP-1R agonist semaglutide [27]. We show that a 4-week treatment with semaglutide pre-stroke in diabetic mice decreased body weight (approximately 20%) and improves glucose metabolism in association with a significant improvement of forelimb grip strength recovery. The paretic forelimb grip strength test assesses the maximal grasping strength (sensorimotor function) of the mice after stroke, mimicking the clinical outcome measure of upper extremity function in stroke patients [28]. Interestingly, semaglutide in combination with BI8271 had a stronger and synergistic effect on weight loss (approximately 33%) and on forelimb grip strength recovery compared with semaglutide monotherapy.

We also used a corridor test to assess impairment of lateralised sensorimotor integration (spatial attention/cognitive function) [29]. In this test, unlike the grip strength test, mice treated with semaglutide exhibited full recovery, reaching levels similar to those of non-diabetic control mice receiving a standard diet. This difference in recovery efficacy between the two tests may be the consequence of various factors. First, the grip strength test is more precise/sensitive to striatal stroke-induced functional impairment and consequently recovery and thus can discriminate small differences between the treatments. Second, the two tests represent different aspects of neurological function. The animals receiving a combination of semaglutide and BI8271 had a significantly larger improvement in both tests compared with those receiving semaglutide monotherapy, suggesting that the extent of post-stroke recovery was positively correlated with the extent of pre-stroke body weight/glucose metabolism normalisation.

Importantly, two sets of results indicate that pre-stroke weight loss by semaglutide or semaglutide/BI8271 was the responsible mechanism behind improved stroke recovery: (1) no additional effect on stroke recovery was achieved by the treatments in the weight-matched studies (Fig. 4); and (2) stroke recovery in the groups treated with semaglutide alone or with semaglutide in combination with BI8271 was entirely independent of acute neuroprotection, as the treatments were withdrawn 2 days pre-stroke and no differences in stroke-induced brain damage were observed between the groups (Fig. 3). This leads us to conclude that there is probably a causal relationship between pre-stroke weight loss and improved stroke recovery.

The metabolic mechanisms specifically responsible for semaglutide-improved stroke recovery are hard to pinpoint because it is essentially impossible to separate the effects of any treatment targeting body weight without also affecting glucose and insulin sensitivity. However, our results show that administration of semaglutide in combination with BI8271 had stronger effects on weight loss (approximately 33%) and on stroke recovery than semaglutide monotherapy. However, even though semaglutide in combination with BI8271 resulted in stronger glycaemic control compared with semaglutide monotherapy, the glycaemic levels in both treatment groups remained below the diabetic threshold (7 mmol/l). These data, together with the fact that acute administration of both semaglutide monotherapy or semaglutide in combination with BI8271 induced the same glycaemic regulation, make us conclude that glycaemic regulation per se may not be crucial in improving stroke recovery. Instead, pre-stroke weight loss is an upstream crucial mechanism in this respect. Importantly, these results suggest that it is possible to reduce stroke disability in diabetes from a preventive perspective by pharmacologically targeting obesity.

The effects of semaglutide on stroke recovery could be further potentiated by NPY2R activation. Indeed, this combined pharmacological strategy is strongly effective in reducing body weight [21–23] and is currently under clinical development for treatment of obesity [30]. Therefore, the potential repositioning of these pharmacological strategies to improve stroke prognosis should be feasible in the future.

No difference in stroke volume was observed between the experimental groups. Moreover, at day 3 post-stroke, all mice showed similar grip strength impairment. This suggests that differences in brain damage were the basis of differences in stroke recovery, but that other mechanisms in the recovery phase explain the improvements in outcome by semaglutide monotherapy or semaglutide in combination with BI8271. The impairment of brain self-repairing mechanisms post-stroke (e.g. neuroplasticity, vascular function and neuroinflammation [31–35]) has been suggested to play an important role in diabetes-induced impaired stroke recovery. However, through which of these mechanisms weight loss improves stroke outcome without a parallel decrease in neuronal loss is undetermined. Based on our recent work showing that diet-induced weight loss improves stroke recovery in association with decreased neuroinflammation, astrocyte reactivity and reduced type 2 diabetes-induced atrophy of parvalbumin-positive interneurons, without reduction of stroke volume [13], we speculate that these cellular mechanisms are also involved after semaglutide/BI8271-induced weight loss. Moreover, semaglutide can protect vascular structures [36] and restore the neurovascular unit [37]. Additionally, studies have shown that modulating functional plasticity [38] or rewiring corticospinal fibres in the intact half of the brain [39] improves post-stroke motor function. Thus, it will be of great interest to investigate in future studies whether weight loss in response to treatment with semaglutide/BI8271 modulates vascular/neuroplasticity processes. In the current study, we present data that potentially advance our knowledge on the additional mechanisms involved. Specifically, we showed an inverse association between serum IGF-1 levels and stroke recovery, in agreement with a clinical study [24], highlighting the potential of determining serum IGF-1 levels at hospitalisation (corresponding to the sub-acute post-stroke IGF-1 levels in our study) as a biomarker for stroke outcome. This is supported by the fact that abnormal IGF-1 levels are associated with insulin resistance [40], which worsens stroke outcome [41]. Moreover, we show that this relationship is already established before stroke.

To determine whether semaglutide monotherapy or semaglutide in combination with BI8271 induced acute neuroprotection in diabetic/obese mice, independently of metabolic regulation, we administered these treatments acutely after reperfusion. We show that semaglutide and BI8271 monotherapies enhanced post-stroke neuronal survival and improved stroke recovery, in line with studies in non-diabetic mice [42–44]. However, we did not detect additive/synergistic effects on acute neuroprotection as a result of combining these treatments. Whether this is because of the limit of detection of the neuroprotection assays, or because the semaglutide-induced neuroprotective effects were already maximal, needs to be investigated further. These results are very important, because GLP-1R agonists have been shown to improve stroke outcome in many preclinical studies in both non-diabetic and diabetic rodent models [45]. However, none of these studies have identified metabolic vs direct neuroprotective effects on stroke recovery. Moreover, these findings are clinically relevant as they clearly show that GLP-1R activation improves stroke recovery by acting on two independent mechanisms: induction of pre-stroke weight loss and acute neuroprotection post-stroke. Although speculative, these findings imply that individuals with type 2 diabetes receiving GLP-1R and/or NPY2R agonists to treat their underlying diseases could possibly profit from both these effects.

Our study has certain limitations. Although we showed that pharmacological induction of weight loss improves stroke recovery in type 2 diabetes, we did not determine the minimal extent of weight loss necessary to improve functional recovery. Moreover, further studies are needed to identify which specific weight loss-induced mediators are causal for stroke recovery, as well as identifying their targets in the brain. Additionally, this study was performed in male mice, and studies in female mice are warranted due to potential differences in the weight loss response after semaglutide/BI8271 between sexes. Finally, stroke especially affects the elderly, and additional studies in older mice should be performed.

In conclusion, the global increase of diabetes [1], as well as the high stroke incidence in individuals with type 2 diabetes [3], are dramatically increasing the medical need for management of post-stroke sequelae. We determined experimentally that pre-stroke weight loss by GLP-1R/NPY2R activation strongly improves stroke recovery in diabetes. This effect may be boosted by additional acute neuroprotective effects of these treatments. Clinical studies are needed to confirm these results. Such studies are feasible as GLP-1R agonists are already in clinical use and NPY2R agonists are under clinical development. Furthermore, GLP-1R agonists can also reduce cardiovascular risk [46]. Therefore, the potential translation of our findings into clinical practice will present an important advantage: individuals living with diabetes/obesity could be treated with a combination of e.g. GLP-1R/NPY2R agonists for body weight regulation and glucose control, potentially resulting in a highly prophylactic value for the management of stroke sequelae.

## Supplementary Information

Below is the link to the electronic supplementary material.

Supplementary file1 (PDF 4747 KB)

## Data Availability

The data that support the findings of this study are not openly available for reasons of sensitivity, but are available from the corresponding author upon reasonable request. Data are located in controlled-access data storage (data repository) at the Karolinska Institutet.

## Abbreviations

GLP-1R: Glucagon-like peptide 1 receptor
HFD: High-fat diet
NeuN: Neuronal nuclear marker
NPY2R: Neuropeptide Y receptor Y2
tMCAO: Transient middle cerebral artery occlusion
