# Supplementary material for: Pre-stroke weight loss by glucagon-like peptide 1 receptor and neuropeptide Y receptor Y2 activation improves post-stroke functional recovery in male diabetic mouse models
Source: Diabetologia. 2025 Oct 15;69(1):230–43. doi: 10.1007/s00125-025-06567-4 (PMC12686085; doi:10.1007/s00125-025-06567-4)
Supplement: Supplementary file 1 — Supplementary file1 (PDF 4747 KB) [file 125_2025_6567_MOESM1_ESM.pdf]

Pre-stroke weight loss by glucagon-like peptide 1 receptor and neuropeptide Y receptor Y2 activation improves post-stroke functional recovery in male diabetic mouse models

Ellen Vercalsteren[1], Dimitra Karampatsi[1], Maria Neicu[1], Mihaela Oana Romanitan[1], Peter Haebel[2], Katherin Bleymehl[2], Thomas Nyström[1], Thomas Klein[2], *Vladimer Darsalia[1], *Cesare Patrone[1].

[1]Department of Clinical Science and Education, Södersjukhuset, Internal Medicine, Karolinska Institutet, Stockholm, Sweden

[2]Boehringer Ingelheim Pharma GmbH & Co. KG, Biberach, Germany

Ellen Vercalsteren (Ph.D.), E-mail ellen.vercalsteren@ki.se, ORCID: 0000-0002-9425-4209

Dimitra Karampatsi (Ph.D.), E-mail dimitra.karampatsi@ki.se, ORCID: 0000-0002-5316-088X

Maria Neicu (M.D.) E-mail maria.neicu@regionstockholm.se

Mihaela Oana Romanitan (M.D., Ph.D.), E-mail mihaela.romanitan@regionstockholm.se, ORCID: 0009-0001-4719-6624

Peter Haebel (Ph.D.), E-mail peter.haebel@boehringer-ingelheim.com, ORCID: 0009-0000-5861-9613

Katherin Bleymehl (Ph.D.), E-mail katherin.bleymehl@boehringer-ingelheim.com, ORCID: 0009-0007-6269-1061

Thomas Nyström (Ph.D., M.D.), E-mail thomas.nystrom@ki.se, ORCID: 0000-0002-3462-7990

Thomas Klein (Ph.D.), E-mail thomas_1.klein@boehringer-ingelheim.com, ORCID: 0000-0001-5762-9166

Vladimer Darsalia (Ph.D.), E-mail vladimer.darsalia@ki.se, ORCID: 0000-0002-6693-934X

Cesare Patrone (Ph.D.), E-mail cesare.patrone@ki.se, ORCID: 0000-0003-0470-4606

CORRESPONDING AUTHORS:

*Vladimer Darsalia (Ph.D.), Karolinska Institutet, Department of Clinical Science and Education, Södersjukhuset, Stockholm, Sweden. Phone: +46 739942702 Fax: +46 (8) 6162933.

*Cesare Patrone (Ph.D.), Karolinska Institutet, Department of Clinical Science and Education, Södersjukhuset, Stockholm, Sweden. Phone: +46 70 4328349 Fax: +46 (8) 6162933.

Abbreviations:

BW	Body weight
IGF-1	Insulin-like growth factor 1
PFA	Paraformaldehyde
T-PBS	Triton X-100 PBS

METHODS

Ethical approval

This study was conducted according to the guidelines of the declaration of Helsinki and has been approved by the regional ethics committee (approval IDs: 1126 and 19666-2022). The work follows the 2010/63/EU directive and is reported according to the ARRIVE guidelines [1].

Diets and Compounds

We used standard diet (D12450K Sniff Spezialdiäten, Germany) and high fat diet with 60% energy from lard fat (D12492, Sniff Spezialdiäten, Germany).

Semaglutide and BI8271 were provided by Boehringer-Ingelheim GmbH & Co. KG, Biberach/Riss, Germany. BI8271 (Ref. Patent WO2022029231, compound 84) is a close analogue of the clinical compound BI1820237 (https://storage.mfn.se/1036dbdb-7fd4-4b0f-a8bc-a6b219b9757f/phase-1-trial-results-from-a-novel-long-acting-npy2-receptor-agonist-in-partnership-between-gubra-a-s-and-boehringer-ingelheim-presented-today-at-the-european-congress-on-obesity-eco-2023.pdf), which is a PYY_{3-36} derived selective NPY2R agonist with a C18 diacid containing half-life extension group attached to the side chain of a lysine residue in position 7.

Metabolic tests

Fasting glycose was measured with a glucometer via blood collected from a tail tip puncture after overnight fasting.

For insulin tolerance tests (ITT), mice were fasted for 2h. Hereafter, baseline glucose levels were measured. Then, the mice were injected i.p. with 0.4 U/kg human insulin and blood glucose levels were measured at 15, 30, 45, 60, 75 and 90 min after injection. The area under the curve was computed for statistical analysis.

Transient middle cerebral artery occlusion

Stroke was induced by tMCAO using the intraluminal filament technique as described previously [2]. Briefly, mice were anesthetized by inhalation of 3% isoflurane and throughout surgery, anaesthesia was maintained by 1.5% isoflurane. Using a heated pad with feedback from a thermometer, body temperature of animals was kept at 37–38 °C. Left external and internal carotid arteries were exposed and a 7–0 silicone-coated monofilament (total diameter 0.17 - 0.18 mm) was inserted into the internal carotid artery until the origin of the MCA was blocked. The occluding filament was removed after 35 minutes. Cerebral blood flow in the vicinity of MCA was monitored by Laser Doppler Blood Flow Monitor (Moor Instruments Ltd, UK), and no differences between the groups were observed (data not shown). Stroke induction was considered unsuccessful when the occluding filament could not be advanced within the internal carotid artery beyond 7-8 mm from the carotid bifurcation, or if mice lacked symptoms of neurological impairment based on the neurological severity score [3] . After surgery, all mice were given soft food and analgesic (Carprofen, 5 mg/ kg) to minimize pain and suffering and were also closely monitored and under veterinary observation.

Assessment of stroke recovery (primary outcome)

Grip strength test

To assess functional recovery after stroke, forelimb grip strength was tested as previously described [4]. Briefly, mice were held firmly by the body and allowed to grasp the grid with the paretic (right) forepaw. Hereafter, they were dragged backwards until their grip was broken. Grip strength was measured using a grip strength meter (Harvard apparatus, MA, USA) at 3 days and 1-4 weeks after tMCAO. Ten trials were performed, and the highest values were used for statistical analysis.

Corridor test

The corridor test was performed in a 50 cm long, 4 cm wide and 15 cm high Plexiglas corridor to assess lateralized sensorimotor integration [5] at 1 and 4 weeks after tMCAO. Mice were fasted ON and on the day of testing, they were habituated in an empty corridor for 2 min. Then, animals were immediately transferred to an identical corridor with 16 pots on each side, each pot containing a flavoured treat. The number of explorations made to the left and right was counted for 5 min. The ratio of right to left explorations was calculated and used for statistical analysis. To calculate the specific amount of recovery for each animal, the difference between week 1 and week 4 (Δ) was calculated for each mouse and used for statistical analysis. All behavioural tests were performed by experimenters blinded for the experimental groups, although this was not always possible due to visible weight differences.

Immunohistochemistry

After 4h of fasting, mice were anesthetized using an overdose of sodium pentobarbital. Hereafter, blood was collected via cardiac puncture and mice were perfused transcardially using PBS followed by a 4% ice-cold paraformaldehyde (PFA) solution. Brains were harvested and stored overnight in 4% PFA at 4°C. After 24 hours of fixation, brains were transferred to PBS containing 25% sucrose and stored at 4°C until they sank. Then, 30-µm thick coronal sections were cut using a sliding microtome, and sections were stored at -20°C in anti-freeze solution.

Brain tissue staining was performed using the free-floating method. Briefly, brain sections were washed in PBS followed by a 20-min incubation in PBS containing 3% H_2O_2 and 10% methanol to quench endogenous peroxidases. Then, sections were washed in PBS and incubated 48h at 4°C in a PBS solution containing 0.25% Triton-X-100 (T-PBS), 3% normal serum and mouse-anti NeuN antibody, a neuronal marker (1:500; #MAB377, Millipore, Burlington, MA, USA; RRID:AB_2298772). Hereafter, sections were washed with T-PBS and

incubated for 2h at RT in a T-PBS solution containing 3% normal serum and biotinylated horse-anti mouse antibody (1:200; #BA-2000, Vector Laboratories, Newark, CA, USA; RRID:AB_2313581). After the secondary antibody incubation, sections were incubated with avidin-biotin complex according to manufacturer's instructions (Vectastain Elite ABC kit, Vector Laboratories), followed by visualization by DAB.

Ischemic stroke volume assessment

In *Study 1* and *2*, ischemic volume was measured by experimenters blinded to treatment groups using the StereoInvestigator software suit, (MBF Bioscience, USA). Briefly, NeuN-labelled serial sections containing visual ischemic damage (See Figure 3F for representative images) were displayed live on a computer monitor using a 1.25x lens. The *v*olume of the whole contralateral, non-damaged hemisphere, and of the intact part of the ipsilateral, stroke-damaged hemisphere was determined using the Cavalieri Estimator method [6]. The ischemic volume was then determined by subtracting the intact ipsilateral volume from the contralateral volume, thus adjusting for stroke-induced tissue shrinkage at 4 weeks after tMCAO. Animals with non-assessable stroke, due to unclear borders of the ischemic lesion, or patchy cell loss that made accurate stroke delineation difficult, or hemorrhages, were excluded in this analysis: *Study 1:* 2 animals in SD, 1 animal in HFD, 2 animals in HFD-Y, 3 animals in HFD-S and 4 animals in HFD-SY; *Study 2:* 2 animals in HFD, 3 animals in HFD-SY, 2 animals in HFD-WM. In *Study 3*, stroke volume was determined by measuring the infarct volume using the Cavalieri Estimator probe. Animals with non-assessable stroke, as described above, were excluded from this analysis: 2 animals in HFD-S, 3 animals in HFD-Y, 1 animal in HFD-SY.

The neuroprotective efficacy of the treatments was further determined by quantifying surviving neurons based on NeuN staining using the optical fractionator method [7] within striatum and cortex on six consecutive serial sections covering the brain volume between bregma 1.5mm

and -0.5mm. Since this method does not rely on the delineation of the ischemic lesion, all samples were included in this quantification.

ELISA immunoassays

Pre-stroke serum insulin levels were determined according to manufacturer's instructions (90080, CrystalChem), using 5 µL of sample.

Secondary outcomes: Serum levels of IGF-1 were quantified before stroke, at 10 days (subacute phase) and at 4 weeks after stroke (endpoint), according to manufacturer's instructions (MG100, R&D Systems, MN, USA), using 10 µL of sample. All ELISA assessments were performed on serum samples pooled from 2-3 animals.

Statistical analysis

Data were checked for statistical outliers by using the ROUT method, and for normality by using the Shapiro-Wilk normality test.

Welch's t-test was used to analyze pre-treatment BW, fasting glycemia and AUC of insulin tolerance test. Brown-Forsythe and Welch ANOVA followed by two-stage linear step-up procedure Benjamini, Krieger and Yekutieli was used for pre-stroke BW, percentage weight change, fasting glycemia, serum insulin, serum IGF-1 and AUC of insulin tolerance test, endpoint grip strength and R/L lateralization in *Study 1*, grip strength 1 week after stroke in *Study 2*, and stroke volume in *Study 1* and *Study 2*. For behavioural analyses and acute glycemic effects of the treatments, two-way repeated measures ANOVA was used followed by either two-stage linear step-up procedure Benjamini, Krieger and Yekutieli or Fisher's LSD test. Pearson correlation was used to analyze correlation between AUC of grip strength and serum IGF-1 levels at different time points in *Study 1*.

All data were analyzed by GraphPad Prism Version 10.0. Data are expressed as mean ± SD. *P*-values less than 0.05 were considered statistically significant.

Sample size calculation

Group sizes were determined based on ≈ 20 % effect size between groups in functional recovery with α = 0.05 and a statistical power of 90%. The standard deviation that was used for sample size calculation was obtained from pilot experiments. Analyses suggested a final sample size of minimal n = 5 per group. After taking into consideration the success rate of stroke surgery, mortality and likelihood of statistical outliers, sample size for groups subjected to stroke was set at n = 15 per group for *Study 1*, n = 11-12 per group for *Study 2*, and n = 10 per group for *Study 3*. The sham group size in *Study 1* was set at n = 5 per group. See ESM Figure 1 for details on the sample size for each analysis in *Study 1, 2* and *3*.

Animals before and after exclusion

205 male C57BL/6J mice (Janvier labs, France) were used in this project. Mice were housed in groups of 5 mice/cage, in environmentally controlled conditions (22 ± 0.5 °C, 12/12 hours light/dark cycle with *ad libitum* access to food and water). Animals were kept under pathogen free conditions in type III size individually ventilated cages with wood chip bedding and nest material. Our mouse model nicely recapitulates the unfolding of type 2 diabetes after obesity (with hyperglycemia and IR) and is therefore suitable for weight loss interventions.

In *Study 1*, Two mice in the HFD-group died during the dietary period due to reasons unrelated to the experiment. 9 mice were removed after tMCAO because of death or reaching the humane endpoint; 2 mice in the SD group, 2 mice in the HFD group, 1 mouse in the HFD-S group and 3 mice in the HFD-Y group. In addition, 1 mouse in the HFD-SY group died during sham surgery.

In *Study 2*, 2 mice in the HFD group died during the tMCAO procedure. Moreover, in the HFD-WM group, data from 1 animal was excluded from analysis since the animal did not perform the test.

In *Study 3*, 5 mice were euthanized shortly after surgery because the humane endpoint was reached: 2 mice in the HFD group, 1 mouse in the HFD-S group, and 2 mice in the HFD-SY group.

RESULTS

Weight loss does not affect grip strength before stroke

To determine whether weight loss by semaglutide or semaglutide+BI8271 treatment affected the grip strength independently of stroke, we measured it before inducing stroke. The results in Figure S3 show that the grip strength was within similar range in all groups, irrespective of weight or metabolic status.

FIGURES

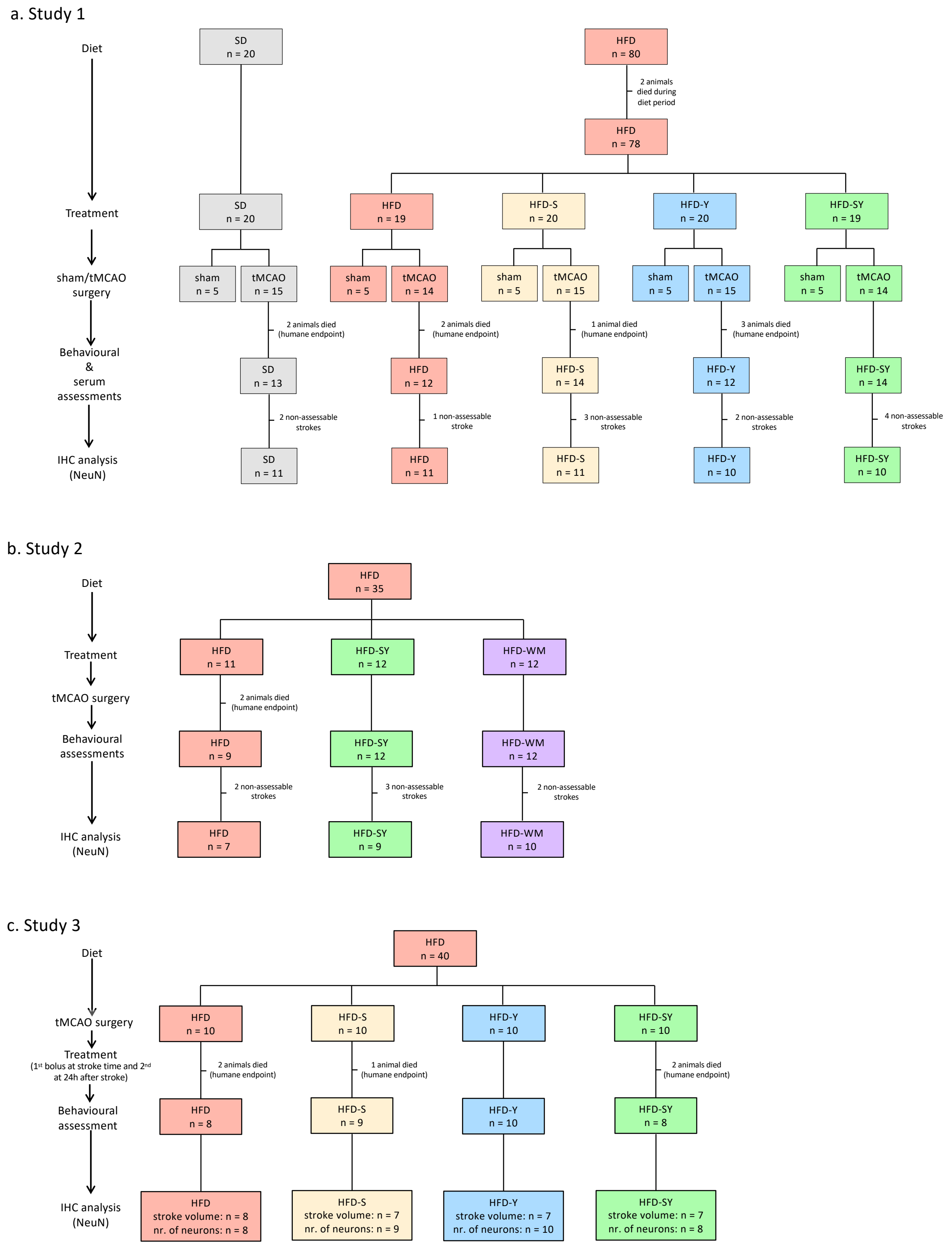

ESM Fig 1. ***flow chart of sample sizes per study.*** Schematic overview of the sample sizes at each phase of the experiment (dietary period, treatment period, sham/tMCAO surgery, behavioural and serum assessments and IHC analysis for stroke volume for *Study 1* (a), *Study 2* (b), and *Study 3* (c). SD = standard diet, HFD = high-fat diet, HFD-S = HFD + semaglutide monotherapy, HFD-Y = HFD + NPY2R BI8271 monotherapy, HFD-SY = HFD + semaglutide and NPY2R BI8271 combination therapy, HFD-WM = HFD + weight matched to weight loss induced by semaglutide and NPY2R BI8271 combination therapy via switch to SD.

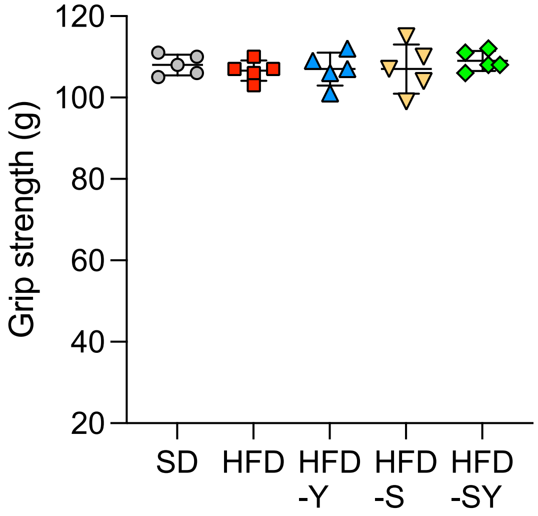

ESM Figure 2. Weight loss does not affect grip strength. Two-way ANOVA followed by Fisher's LSD test.

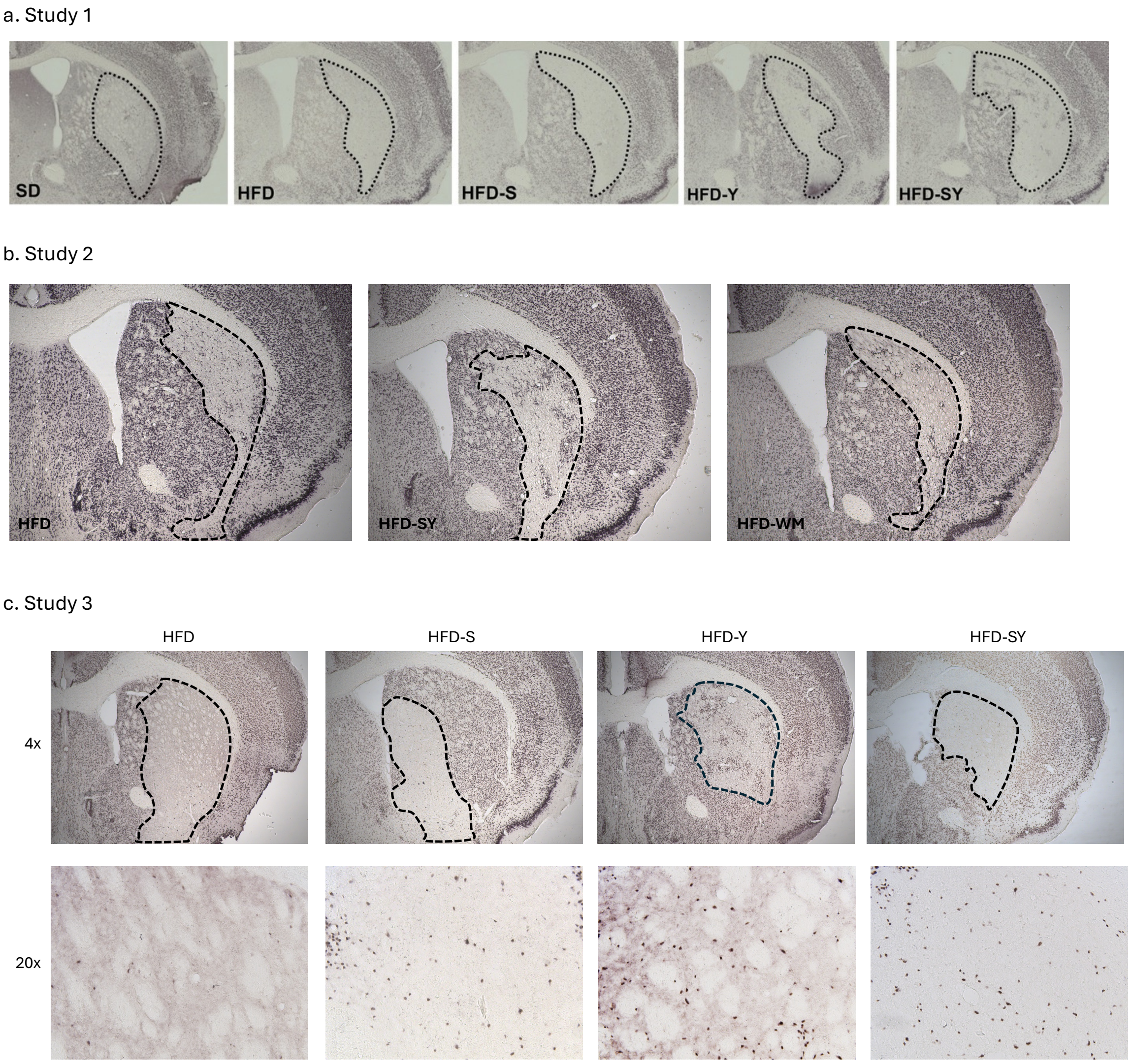

ESM Figure 3. Representative images of the stroke volumes from all treatment groups in (a) Study 1, (b) Study 2 and (c) Study 3. The dotted line delineates the infarct area, the bottom row (20x) in (c) depicts surviving neurons inside the infarct site.

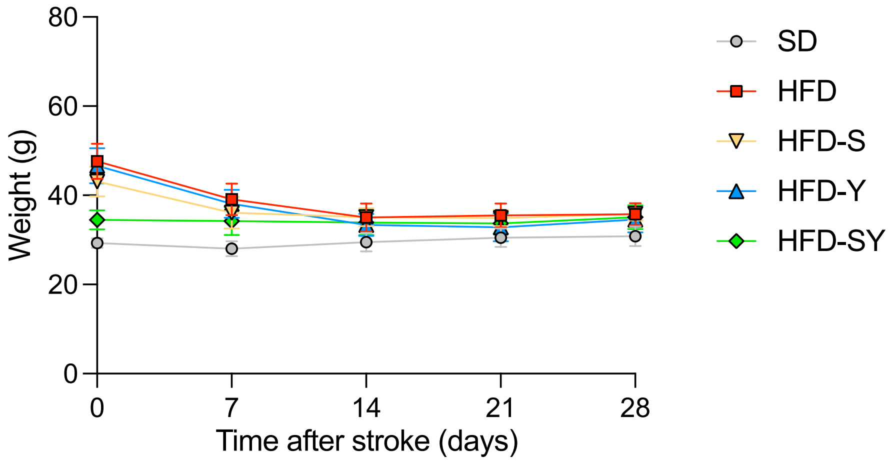

ESM Figure 4. Post stroke weight loss

REFERENCES

[1] Percie du Sert N, Hurst V, Ahluwalia A, et al. (2020) The ARRIVE guidelines 2.0: updated guidelines for reporting animal research. J Physiol 598(18): 3793-3801. 10.1113/JP280389

[2] Hara H, Friedlander RM, Gagliardini V, et al. (1997) Inhibition of interleukin 1beta converting enzyme family proteases reduces ischemic and excitotoxic neuronal damage. Proc Natl Acad Sci U S A 94(5): 2007-2012. 10.1073/pnas.94.5.2007

[3] Bederson JB, Pitts LH, Tsuji M, Nishimura MC, Davis RL, Bartkowski H (1986) Rat middle cerebral artery occlusion: evaluation of the model and development of a neurologic examination. Stroke 17(3): 472-476. 10.1161/01.str.17.3.472

[4] Karampatsi D, Zabala A, Wilhelmsson U, et al. (2021) Diet-induced weight loss in obese/diabetic mice normalizes glucose metabolism and promotes functional recovery after stroke. Cardiovasc Diabetol 20(1): 240. 10.1186/s12933-021-01426-z

[5] Wattananit S, Tornero D, Graubardt N, et al. (2016) Monocyte-Derived Macrophages Contribute to Spontaneous Long-Term Functional Recovery after Stroke in Mice. J Neurosci 36(15): 4182-4195. 10.1523/JNEUROSCI.4317-15.2016

[6] Rosen GD, Harry JD (1990) Brain volume estimation from serial section measurements: a comparison of methodologies. Journal of neuroscience methods 35(2): 115-124. 10.1016/0165-0270(90)90101-k

[7] Gundersen HJ, Bendtsen TF, Korbo L, et al. (1988) Some new, simple and efficient stereological methods and their use in pathological research and diagnosis. APMIS 96(5): 379-394. 10.1111/j.1699-0463.1988.tb05320.x
